# Measuring plant biomass remotely using drones in arid landscapes

**DOI:** 10.1002/ece3.8891

**Published:** 2022-05-13

**Authors:** Justin A. McCann, David A. Keith, Richard T. Kingsford

**Affiliations:** ^1^ Centre for Ecosystem Science School of Biological, Earth and Environmental Sciences UNSW Sydney New South Wales Australia; ^2^ 591183 Bush Heritage Australia Melbourne Victoria Australia

**Keywords:** allometry, biomass, drone, plant, unmanned aerial system, unmanned aerial vehicle

## Abstract

1. Measurement of variation in plant biomass is essential for answering many ecological and evolutionary questions. Quantitative estimates require plant destruction for laboratory analyses, while field studies use allometric approaches based on simple measurement of plant dimensions.
2. We estimated the biomass of individual shrub‐sized plants, using a low‐cost unmanned aerial system (drone), enabling rapid data collection and non‐destructive sampling. We compared volume measurement (a surrogate for biomass) and sampling time, from the simple dimension measurements and drone, to accurate laboratory‐derived biomass weights. We focused on three Australian plant species which are ecologically important to their terrestrial and floodplain ecosystems: porcupine grass *Triodia scariosa*, Queensland bluebush *Chenopodium auricomum*, and lignum *Duma florulenta*.
3. Estimated volume from the drone was more accurate than simple dimension measurements for porcupine grass and Queensland bluebush, compared to estimates from laboratory analyses but, not for lignum. The latter had a sparse canopy, with thin branches, few vestigial leaves and a similar color to the ground. Data collection and analysis consistently required more time for the drone method than the simple dimension measurements, but this would improve with automation.
4. The drone method promises considerable potential for some plant species, allowing data to be collected over large spatial scales and, in time series, increasing opportunities to answer complex ecological and evolutionary questions and monitor the state of ecosystems and plant populations.

## Graphical abstract

We estimated the biomass of individual shrub‐sized plants non‐destructively, using a low‐cost drone. We compared volume measurement (a surrogate for biomass) and sampling time, from an existing manual field measurement technique and the drone, to accurate laboratory‐derived biomass weights. The drone method promises considerable potential for some plant species, allowing data to be collected over large spatial scales and, in time series, increasing opportunities to answer complex ecological and evolutionary questions and monitor the state of plant populations.

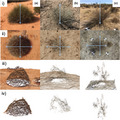

## 1 INTRODUCTION

Biomass of plant communities reflects evolutionary (Berner et al., 2018) and ecological drivers (Westcott et al., 2014), influenced by direct (Friedel et al., 2003; Yelenik & D’Antonio, 2013) or indirect (McIntyre et al., 2015) anthropogenic pressures. Measurement of biomass can help identify changes in states and processes of ecosystems, but data collection is often intensive and time‐consuming, limiting large spatial coverage (Ferrier, 2012; Nichols & Williams, 2006). Ecological monitoring surveys often require biomass estimation for individual plants, rather than the more common biomass per area estimation that is used in agricultural production (Proulx et al., 2015). There are two main approaches to measuring vegetation biomass: direct measurements of plants in the field (Catchpole & Wheeler, 1992), supported by laboratory analyses, or remote sensing using either aerial photography, satellite imagery, radar or light detection and ranging (LiDAR) to estimate biomass with reflectance indices (Peng et al., 2019), cover or structural information (Kumar & Mutanga, 2017).

Simple measurement of plant volume in the field is often used as a surrogate for biomass (Proulx et al., 2015), by estimating height and two perpendicular width measurements, providing a convex hull for individual plants (Bonham, 1989). Plant volume may also be estimated from photographs or quantitative relationships between cover and height, varying with age and species of plant (Catchpole & Wheeler, 1992; Westcott et al., 2014). Such indirect measures efficiently sample plant structure and volume, but are limited to measuring overstorey vegetation (Suganuma et al., 2006). Laboratory measures of biomass are most accurate, but involve destructive removal of the whole plant then oven drying and weighing to estimate dry weight biomass (Bonham, 1989).

Remotely sensed imagery is also increasingly used to estimate above‐ground biomass, over long temporal periods, at continental and global scales (Lu, 2006), but this approach has significant limitations. Estimates focus on monocultures in agricultural and forestry contexts (Kumar & Mutanga, 2017), incorporating phenological stage information of the plantation to increase accuracy (Peng et al., 2019). Some mapping of ecosystem composition has helped to interpret biomass estimates, but has not been undertaken for complex plant communities or individual plants (Lu et al., 2016), given that the best spatial resolution from satellite remote sensing is about 60 cm (e.g., IKONOS, Quickbird). Airborne LiDAR can measure distance of the sensor from both the ground and leaf canopy using lasers, producing accurate and fine spatial scale remote sensing estimates of vegetation biomass (Zolkos et al., 2013), but at considerable cost (Lu, 2006) and seldom accounting for small branches and leaf canopy biomass (Verschuyl et al., 2018; Zolkos et al., 2013). Terrestrial Laser Scanning (ground‐based LiDAR) can be used to estimate biomass for individual trees (Kankare et al., 2013; Shendryk et al., 2016) but is time‐consuming for stationary equipment, particularly in remote areas and steep terrain. Mobile equipment generates complex data, limiting application in temporal vegetation surveys, particularly of individual plants. There is a need to identify the efficacy of this technology for measuring individual plant biomass in ecological surveys, recognizing that it will not necessarily replace field surveys unless it is scalable.

More recently drones are used to collect remotely sensed data at low cost (Anderson & Gaston, 2013). Drone‐based methods utilize Structure from Motion (SfM) techniques to create three‐dimensional (3D) point clouds, typically predicting the volume of a solid object (Dandois & Ellis, 2010). Development of SfM techniques has predominantly focused on industry such as precision agriculture (Torres‐Sánchez et al., 2015), but they are increasingly useful for mapping natural vegetation communities (Cruzan et al., 2016), including biomass of leaf litter in Australia (Wallace et al., 2017) and shrubs in semi‐arid United States (Cunliffe et al., 2016). Developments in automating data collection, processing, and analysis could potentially provide data relevant for quantifying variation in plant size among species.

Despite this promise, estimates are often based on fewer data than manual methods, using only height (Cunliffe et al., 2021). Often the accuracy of vegetation biomass estimates from drones is poorly known. We aimed to estimate dry weight biomass of three plant species with contrasting growth forms (porcupine grass *Triodia scariosa*, Queensland bluebush *Chenopodium auricomum*, and lignum *Duma florulenta*) in the mid stories of semi‐arid woodlands, using drone‐collected data. The species occupy different landscape settings (floodplain, terrestrial) in semi‐arid zone plant communities. Our objective was to compare drone‐based estimates of dry weight biomass and their time costs for these species, with those based on simple dimension measurements and laboratory analyses.

## 2 MATERIALS AND METHODS

### 2.1 Field sampling

We collected biomass data in two locations in semi‐arid Australia: Mallee Woodlands (33° 24^’^ S, 141^o^ 10^’^ E), sampled in Spring (October 2017) and North‐west Floodplain Woodlands (29^o^ 15^’^ S, 145^o^ E), sampled in Autumn (April 2017). The former comprised low woodlands of mallee trees (ridge‐fruited mallee *Eucalyptus costata* subsp. *murrayana*, white mallee *E*. *dumosa*, and red mallee *E*.* socialis*), dispersed with cypress pines *Callitris verrucosa*, semi‐sclerophyl shrubs (*Acacia*, *Beyeria*, *Triodia* and *Vittadinia* genera) and a discontinuous hummock grass layer of porcupine grass (Keith, 2004; Yates et al., 2017). The second plant community comprised an open canopy of floodplain eucalypts (yapunyah *E*. *ochrophloia*, coolabah *E*. *coolabah*, and black box *E*. *largiflorens*), a sparse shrub layer of lignum, Queensland bluebush and a continuous grassy ground cover, including rat's tail couch *Sporobolus mitchellii*, Warrego summer grass *Paspalidium jubiflorum* and purple lovegrass *Eragrostis lacunaria* (Catford et al., 2017; Hunter & Hunter, 2016; Keith, 2004). The floodplain woodland had variable grass height surrounding the targeted mid‐story vegetation.

We defined three size classes for our three mid‐story species (Table 1), representing typical structure in the field, to ensure that the method captured the full range of plant sizes of each species. As well as the intrinsic value of the species in their ecosystems, we selected them because they are functionally important to animal species. On the Dune Mallee Woodlands, we selected the perennial domed hummock forming porcupine grass, given its importance for fire management (Bradstock & Gill, 1993; Wright & Clarke, 2007) and value as cover for small vertebrates (Menkhorst & Bennett, 1990). The North‐west Floodplain Woodlands included Queensland bluebush and lignum. Queensland bluebush is a compact to open‐canopied shrub targeted by floodplain grazing (Capon, 2003) and lignum is a wiry shrub with sparse foliage, functionally important as habitat for waterbird breeding colonies on wetlands (Brandis et al., 2011).

**TABLE 1:** Mean estimates (±SE) of volumes of plants estimated using simple dimension measurements and drone measurements and wet and dry weight biomass from laboratory analyses for three individuals from three different size classes of three plant species from semi‐arid Australia

Species	Size class	Simple dimension volume (m^3^)	Drone volume (m^3^)	Laboratory analysis	
				Wet biomass (g)	Dry biomass (g)
Queensland bluebush	Small (2–10 cm high)	0.0009 (0.0004)	0.0005 (0.0004)	16.6 (6.93)	8.93 (3.75)
	Medium (11–23 cm high)	0.0019 (0.0005)	0.0006 (0.0004)	22.2 (5.15)	13.9 (3.34)
	Large (24–73 cm high)	0.107 (0.0070)	0.217 (0.0496)	681 (88.1)	525 (71.7)
Lignum	Small (5–20 cm high)	0.0008 (0.0003)	0.0001 (0.0000)	10.1 (1.22)	6.26 (0.809)
	Medium (21–53 cm high)	0.0178 (0.0033)	0.0096 (0.0055)	41.0 (2.96)	21.8 (2.01)
	Large (59–137 cm high)	2.10 (0.256)	3.17 (0.804)	6350 (1030)	4570 (766)
Porcupine grass	Small (30–40 cm high)	0.0174 (0.0045)	0.0395 (0.0134)	503 (196)	428 (166)
	Medium (40–50 cm high)	0.0303 (0.0055)	0.0763 (0.0176)	120 (211)	1060 (192)
	Large (50–76 cm high)	0.123 (0.0311)	0.275 (0.0582)	376 (519)	3330 (491)

We estimated dry weight biomass by measuring volume with two field methods: a simple dimension measurement and a drone. Volume was not directly comparable between methods, as the drone method detected detailed structure, not simple dimensions, and so we harvested samples destructively to quantify dry weight biomass. We randomly stratified sampling using each size class and species’ combination, ensuring individuals (*n* = 3) were under full sunlight and in good health, representative of most individuals in the field.

**FIGURE 1:**
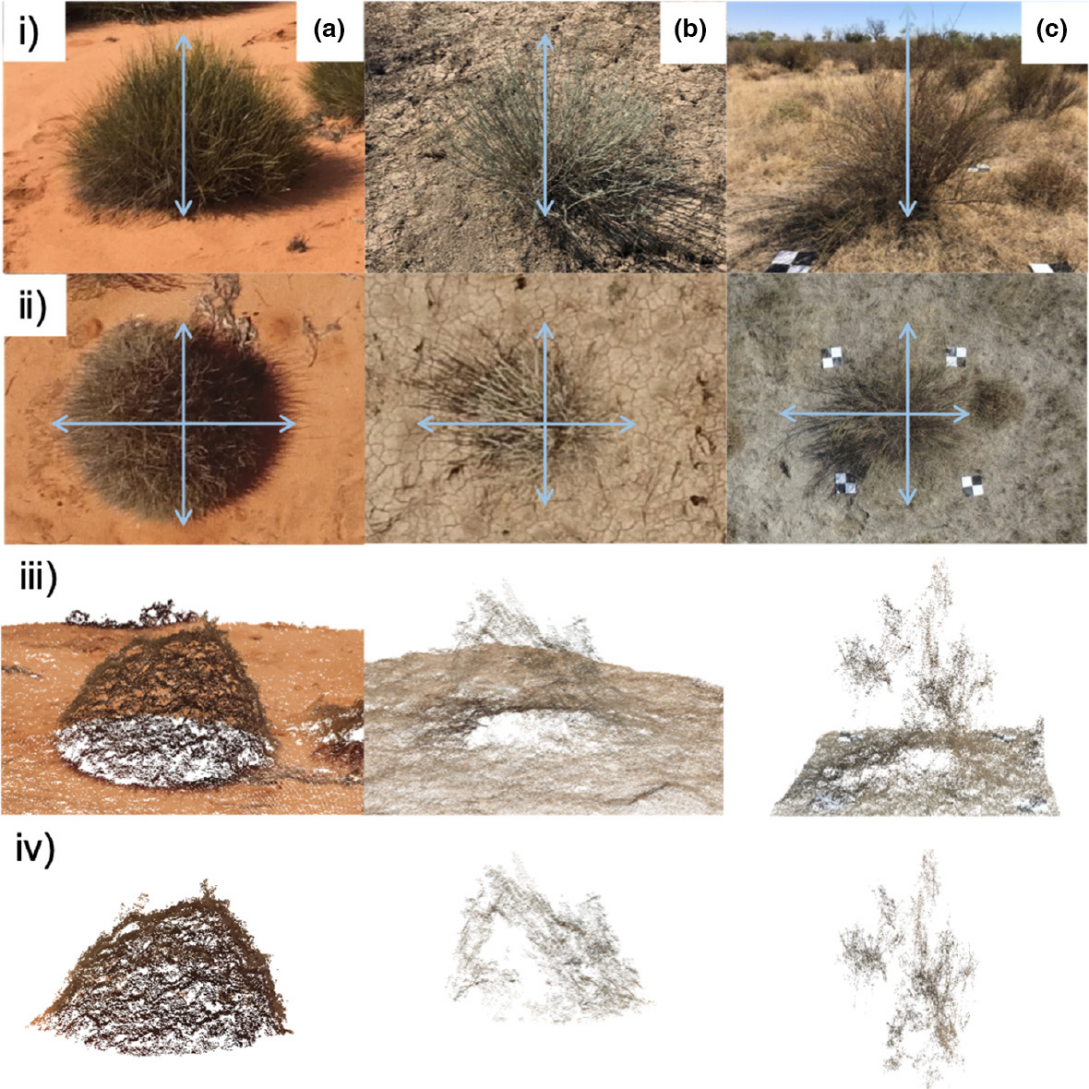
Measurement of an individual from three species of semi‐arid plants (a) porcupine grass (b) Queensland bluebush and (c) lignum species, showing for each: (i) height and (ii) two width measurements for simple dimension measurements, which were measured with a field with a ruler; (iii) the resulting point cloud from the drone image, after processing in Pix4D and; (iv) the point cloud after manual removal of nearby vegetation in CloudCompare. Lignum features scale constraint markers spaced 2 m apart. Other species used markers that have been cropped out to focus on the smaller plant size

For simple dimension measurements, we measured height from ground level to the tallest plant part and crown circumference, using the longest horizontal dimension of the plant and its perpendicular axis to produce a 3D octahedron. This allowed estimation of volume (see Bonham, 1989). We then surveyed each individual plant, using a DJI Phantom 3 professional drone (DJI, Shenzhen, China) with its standard mounted camera (12 megapixel (MP) camera, fixed lens and focal length, mounted with a stabilizing unit). Ground control points of known dimensions were placed for each plant, to generate two perpendicular scale constraints, increasing the accuracy of the resulting point cloud (Figure 1). We flew a manually navigated grid pattern at 10 m above ground and within 3 h of solar midday to minimize shadows, using a combination of downwards (nadir) and angled (non‐nadir) images, with at least 70% overlap of each image. Where plants were close together, multiple plants were surveyed in one flight. The elevation provided about 40 high‐resolution images (<1 mm ground sample distance) for each plant, recorded as red, green and blue (RGB) jpeg files in the visible spectrum (Figure 1b). Our methods were similar to those used to estimate biomass with drone photogrammetry in a global experiment (Cunliffe & Anderson, 2019), except we used a low‐cost consumer‐level drone (not survey‐grade equipment), and relative space (not precision GPS).

After collecting field measurements, we destructively sampled each plant for laboratory measurements of dry and wet biomass by harvesting all above‐ground plant matter. Plants were stored in plastic bags with moist paper towels for transport. Subsequently, wet weight biomass of each plant was measured (stems and leaves amalgamated) before drying it in an oven (70°C for at least 72 h), after which dry biomass was weighed (Pérez‐Harguindeguy et al., 2013). There was a very strong relationship between wet (in‐field) and dry weight measurements of biomass among any of the three species (log dry weight = −0.31 + 1.06 log wet weight, *R* ^2^ = .99, *p* < .01, Appendix Figure S1).

### 2.2 Drone image analysis

We used SfM (using Pix4Dmapper software, Pix4D SA, 2018) to generate a 3D model of each plant (point cloud, Gross & Heumann, 2016), allowing estimation of volume. Each plant point cloud was set with scale constraints from the ground control points to improve measurement precision (Figure 1). Unconstrained point cloud measurements had an average error of 1.90% (±0.23% SE), compared to point clouds constrained by ground control points and so we used scale constraints to generate a 3D model for each plant, exported as a point cloud (Figure 1). We manually selected each plant from point clouds using CloudCompare (V2.8.1, 2018), ensuring that nearby plants (e.g., grasses) were not included (Figure 1). This step can be omitted where canopy height models delineate individuals from surrounding vegetation (see Cunliffe et al., 2016) but, on the floodplain environment, ephemeral plants significantly masked the ground surface and overlapped with the plants of interest. We exported the point cloud for each plant into the R statistical software environment (R Core Team, 2018) and calculated the minimum convex hull of the plant using *RLiDAR* (Silva et al., 2017), the same measure used in standard in‐field biomass estimation (Bonham, 1989).

### 2.3 Statistical analyses

We compared estimates of plant volume based on simple dimensions and drone‐derived point clouds to our laboratory estimates of dry weight biomass. We fitted a linear model for each species, specifying our estimates of laboratory dry weight biomass as the response variable and separately analyzing relationships with predictor variables of simple dimension and drone measures in the R software environment (R Core Team, 2018). Data were pooled for analyses with size class as a covariable within species to ensure that methods and models were applicable to the whole size range for each species. For Queensland bluebush and lignum data, we log‐transformed and added a square term to ensure that assumptions of normality and variance homogeneity were met; no transformation was required for porcupine grass. We used k‐fold cross validation of each linear model to compare resulting errors and slopes between the estimation techniques, allowing us to identify the estimation technique with the lowest root mean square error (RMSE) for each species. We also compared the amount of time to derive dry weight biomass estimates from the two field methods in the North‐west Floodplain Woodlands (lignum and Queensland bluebush), including capture and processing of images, plant harvesting, computation time, and time spent selecting individual plants from the 3D point cloud.

## 3 RESULTS

There were clear differences between simple dimension and drone measures in representing the complexity of plants (Figure 1). Simple dimension measurements only captured three measures of each plant (height and two width measures, Figure 1); therefore, quite differently shaped plants could have the same volume. This contrasted the complex 3D structures in the form of a point cloud measured using drone imagery (Figure 1). Point clouds captured a truer shape of each plant but relied on detecting thin branches from photographs, a weakness for lignum branches.

The simple dimension measurements for porcupine grass showed relatively invariant estimates of height and width, given the similar facets of the plant. Drone measures reflected this uniformity in form, but the point cloud showed considerable complexity in finer plant detail. Individuals of porcupine grass were uniformly shaped, whereas Queensland bluebush were not. Typically, small plants were narrow and large plants had a round but irregular shape. This was not captured in simple dimension measurements but was apparent in the two‐lobed point cloud shape (Figure 1).

Lignum plants were highly variable in shape, often with thin branches protruding from the main plant form. This resulted in large volume measurements for simple dimension measurements but a relatively low dry weight biomass, compared to the other species. For lignum, the simple dimension measurement of width was longer in one plane than the other in Figure 1. Visual assessment showed that point cloud reconstructions underestimated the true size of lignum plants, not reliably reconstructing plant parts under one centimeter in diameter and inadequately capturing the full plant width (Figure 1).

**FIGURE 2:**
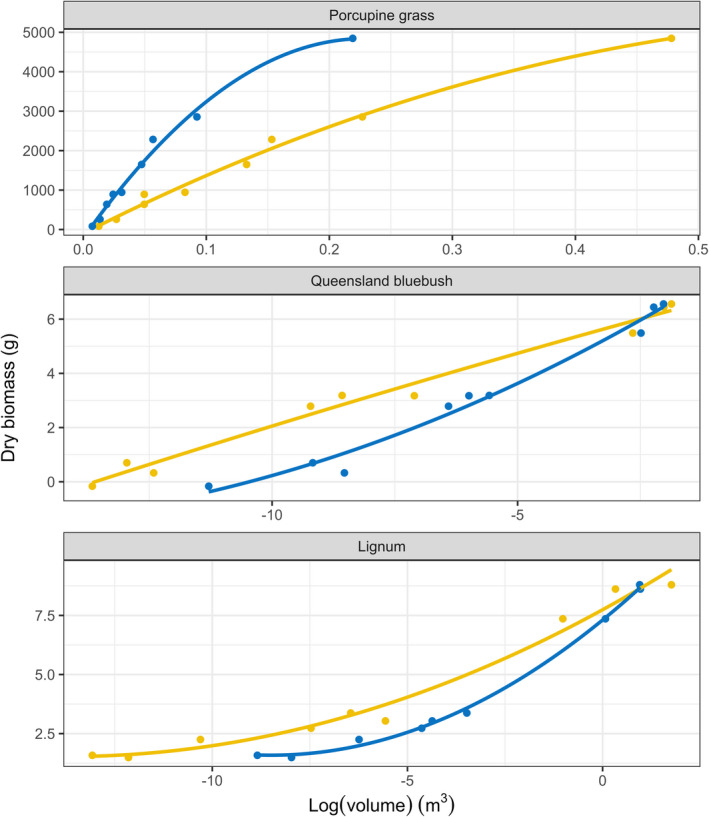
Fitted linear models for three plant species, porcupine grass, Queensland bluebush and lignum and relationships between dry weight biomass, measured in the laboratory and measures of volume, using drone imagery and associated point clouds (yellow) and simple dimension measurements (blue), taken in the field for individuals. Negative volume values for Queensland bluebush and lignum plots reflect logged values

Simple dimension and drone measures of volume for the same plant varied considerably (Figure 2). For porcupine grass, relationship between dry weight biomass and volume estimated from our two measures was considerably different (Figure 2); simple dimension measurement had a steeper and more contracted relationship compared to our drone measure (Figure 2). Our simple dimension measurement and its volumetric surrogate was a useful measure of dry weight biomass, with most of the variance explained (*p* < .01, Table 2, Figure 2). Volume of porcupine grass estimated with the drone method was also a good predictor of dry weight biomass, with the same amount of variation explained by a fitted linear model (*p* < .01, Table 2, Figure 2). The individual with the largest volume was an outlier, influencing both relationships. Examination of the residuals for these two models, across the different size classes, indicated no obvious pattern related to size class of porcupine grass (Appendix Figure S2). Cross validation of these models for porcupine grass showed that the drone method was superior, explaining a higher proportion of variance than simple dimension measurement (RMSE, Table 2).

**TABLE 2:** Summary of linear analyses, including cross validation analyses (root mean square error, RMSE) of separate relationships between our response variable, dry weight biomass (*y* variable), and volume estimates from the simple and drone measure (*x* variables), measured in the laboratory for individual plants of porcupine grass, Queensland bluebush and lignum

Plant species	Technique	Linear relationship	Variance	RMSE
Porcupine grass	Simple dimension measurement	*y* = −218.75 + 44270.61*x* − 96854.22*x* ^2^	0.988	1,332,406
	Drone	*y* = −100.27 + 15810.91*x* − 11429.54*x* ^2^	0.988	31,531
Queensland bluebush	Simple dimension measurement	log(*y*) = 8.72 + 1.19 log(*x*) + 0.03 log(*x*)^2^	0.973	0.344
	Drone	log(*y*) = 7.23 + 0.48 log(*x*)	0.976	0.228
Lignum	Simple dimension measurement	log(*y*) = 7.31 + 1.35 log(*x*) + 0.08 log(*x*)^2^	0.997	0.035
	Drone	log(*y*) = 7.74 + 0.9 log(*x*) + 0.03 log(*x*)^2^	0.970	0.507

Note

Low values in Queensland bluebush and lignum plots are the result of log values in their models. All models were significant (*p* < .001).

For Queensland bluebush, there was a slight difference between the two methods relationships between volume and dry weight biomass, with both models having a similar shaped curve (Figure 2). Our simple dimension measurements explained less variation, with its fitted linear model (*p* < .01, Table 2, Figure 2) than the drone method, which explained more of the variation in dry weight biomass (*p* < .01, Table 2, Figure 2). Examination of residual plots for the models in relation to size classes indicated that mid‐size classed individuals (Table 1) tended to be underestimated for the simple dimension measurements method (Appendix Figure S2). Both methods were good predictors of dry weight biomass for Queensland bluebush. Model cross validation showed that the drone method model performed better than the simple dimension measurement model, explaining a higher proportion of variance (Table 2).

For lignum, both methods explained significant variation in dry weight biomass estimates, with the drone method fitting a flatter curve (Figure 2). The fitted curve can be explained by different partitioning between dense stems and light branches with plant size. Simple dimension measurements were a more accurate predictor of dry weight biomass (*p* < .01, Table 2, Figure 2), explaining more variation than the drone method (*p* < .01, Table 2, Figure 2). Residuals did not show any clear pattern in size classes for lignum (Appendix Figure S2). Model cross validation showed the simple dimension measurement method was a better method for lignum, with a lower RMSE, explaining a higher proportion of the variance of volume (Table 2).

**FIGURE 3:**
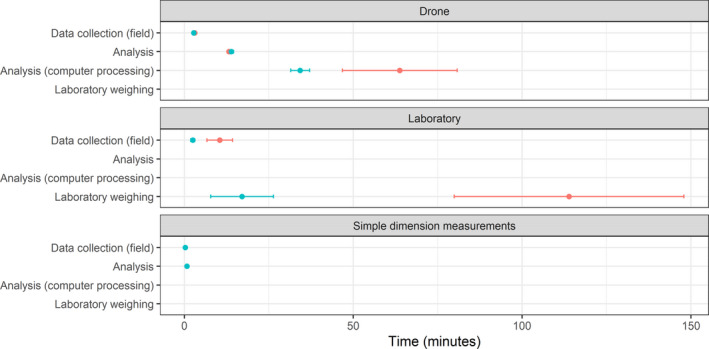
Average data collection and analysis (field, computer, laboratory) time (±SE) for simple dimension (green) and drone (orange) measures and laboratory analysis of nine Queensland bluebush and lignum individual plants in semi‐arid Australia. Laboratory drying took an additional 72 h (4320 min) for both species

Field data collection was the least time‐consuming step for each of the three methods (Figure 3), whereas data processing and analysis were the most time‐consuming tasks. For the drone method, fieldwork took only 4% of overall time, with 75% taken by computer processing which needed no human intervention. The smaller size and closer spacing of sampled Queensland bluebush allowed photographing in two flights, reducing the time taken to land and launch between sampling, resulting in shorter field sampling and image analysis time per plant. The laboratory method was least time‐efficient. Weighing each plant on laboratory scales took 91% of the laboratory method processing time.

For the two species analyzed for time (lignum and Queensland bluebush), there were significant differences between simple dimension and drone measures (analysis of variance, *F*_1,2df_ = 31787, *p* < .01). Simple dimension measurements were the most rapidly collected data for each plant (Figure 3) followed by the drone method. The laboratory analysis took the longest time, with 72 additional hours of oven drying, omitted from this analysis. Time spent measuring in the field was lower for our simple dimension measurements, but our drone flight covered 2500 square meters in 33 min. The time spent collecting data in the field would be similar between the simple dimension measurements and drone measurement if there were 110 plants in this area (density of one plant per ~22.7 square meters). Time spent decreased relatively with the drone method when plants exceeded this density. Plant species had little effect on data collection times for simple dimension measurement and drone measurement, but large lignum plants took longer to harvest and pack for laboratory analysis (25 min ±2.9 SE) than the smaller sized Queensland bluebush (large size class 4.7 min ±0.3 SE). Analysis time was similar for lignum and Queensland bluebush, but computer processing analysis took longer for lignum reflecting the larger plant size (Figure 3).

## 4 DISCUSSION

Drones are a powerful tool for collecting environmental imagery, particularly for identifying landform and structure (Cruzan et al., 2016). Drones clearly showed promise for the three species in effectively estimating dry weight biomass, using volume as a surrogate (Table 2, Figures 1 and 2). Such effectiveness was demonstrated for similar shaped plants and leaf litter in Mediterranean environments (Cunliffe et al., 2016; Wallace et al., 2017), and has been developed for easily measurable ecosystems (Karpina et al., 2016). Before our work, biomass estimated from drone imagery was related to accurate laboratory analysis for area with crops, but not individual plants. Measurement of individual plants has important ecological applications and could be used in studies of plant population dynamics and ecological monitoring surveys, supported by field data. Our drone measures of biomass were not equally effective among the three species and, for small sample sizes, were more costly in terms of time than simple dimension measurements. When sample sizes exceed 110 plants and density exceeds one plant per ~22.7 square meters, however, drone measures of biomass become more efficient for the time spent in the field. Our drone imagery also provided information on structure and form of vegetation that could be further analyzed.

Non‐destructive measures are required in projects analyzing temporal patterns in states and processes of ecosystems, measuring ecosystem health (McIntyre et al., 2015), and identifying ecological processes (Moukomla et al., 2018). Nonetheless, some destructive sampling to quantify allometric relationships for calibration is desirable if absolute values rather than comparative of biomass are required. Simplification of large scale biomass monitoring will help address the shortfall in long‐term monitoring of such ecological characteristics (Belovsky et al., 2004). The absence of measurement bias across size classes (Figure 3) indicates potential value to use drone imagery to track individual plant changes reliably over time. This relative comparison could have wide application for monitoring outcomes of ecosystem restoration and measuring impacts of disturbances. For example, estimating biomass after fire could inform fire management strategies (Brown et al., 2009). Further, large areas of the semi‐arid zone are overgrazed (Eldridge & Delgado‐Baquerizo, 2017) and the drone method could track associated biomass and structure in response to grazing. There are also opportunities to track effects of climate change (Berner et al., 2018), insect damage (Stone & Coops, 2004), and disease (Reiter et al., 2004) on vegetation communities.

Our drone method also captured detailed plant structure, in the plant convex hull, compared to simple dimension measurements which assume an octahedron (Figure 1). The dense porcupine grass provided the best correspondence with biomass for both simple dimension and drone measurement (Figure 2). The other two species, Queensland bluebush and lignum, had less dense canopies (Figure 1), which lost leaves during dry periods (Freestone et al., 2017) further reducing canopy density thus measurement accuracy (Figure 2). The drone method was no better than the simple dimension measure for lignum. It is difficult to measure canopy volume for this shrub regardless of the method used, including LiDAR, because it has a habit of fine, long stems with few, small leaves (Capon et al., 2009), making assumptions for biomass calculation unreliable. These traits are not easily resolved in image processing because the fine stems are a similar color to the ground. The drone method shortcomings could be offset by flying at a lower altitude to capture adequate image detail to delineate branches, or using a higher camera resolution. Structure information collected with the drone method may be important beyond biomass estimation. For lignum, structural density is important for its value as nesting habitat for waterbirds (Brandis et al., 2011), which is missed in simple dimension estimates of biomass. Additional products of the SfM procedures used in the drone method are orthorectified imagery and digital terrain models, which are useful for landscape vegetation structure, patch analysis, vegetation mapping, and visualizing plant condition.

The model relating dry weight biomass to volume could also be calibrated to favorable or poor growth conditions, or to other species by resampling biomass (Bonham, 1989), making the drone method applicable to plants species discernible as an individual from above. Published allometry values could then be used in other applications. It could be extended to shrubs and grasses that have a similar habit to the species measured, with sparse overstoreys, such as shrubs in Australia's alpine and heathland areas, and to hummock grasslands, as well as other ecosystems with scattered trees and open woodland worldwide, such as alpine woody shrublands and Mediterranean open woodlands (Cunliffe et al., 2016; Nie et al., 2016). The drone method was most suited to high‐density growth forms, such as the structurally consistent porcupine grass which did not obscure information relevant to biomass and species with distinct color differences to underlying substrate. Trees have more variation in tissue densities and their canopies can obscure an absence of leaves and branches lower on the plant, making them currently less well suited to this method, requiring technology such as LiDAR to “see through” the tree canopy to measure trunk volume and inconsistent structure.

Drone datasets can be re‐analyzed with improved computation methods, improving data with future developments. Potentially improved resolution and machine learning algorithms could be applied and allow for increasing accuracy of biomass for a range of vegetation communities. Additional to these readily applicable improvements, advances in drone technology and automated shape recognition may further improve accuracy. As consumer‐grade drone cameras increase in resolution above 12 MP, finer plant parts will be resolved in images, reducing error in the dry weight biomass to volume relationship. Further, automatic shape recognition and separation of the plant in the 3D model from the ground surface will increase efficiency as it has for 3D airborne laser scanning data (Shendryk et al., 2016), increasing the potential for automated data processing and analysis for species identification and size variation.

Regardless of the method, biomass must be measured in the laboratory to develop an allometric relationship for calibration. The drone and simple dimension estimates will both have the same cost for this survey establishment, but will comparatively reduce with the scale of study. The drone method would therefore become comparatively more efficient with greater survey size. There are also likely to be efficiencies in data collection and processing. The drone method was more time‐consuming but required less effort than the simple dimension measurement (Figure 3). This is likely to significantly improve. First, by sampling many plants in each flight, as the longest time was taken when the drone was separately launched for each plant. Second, structuring automatic flight plans can be more efficient than manual piloting. Finally, the number of markers required to delineate scale may be reduced by sampling plants close together, sharing scale constraints, and by utilizing equipment with precise positioning (GPS error correction) which reduces scale constraints required for each 3D model. Improvements in automation could reduce manual labor in selecting plants from point clouds, reducing processing time for the drone method. More automated drone methods also have potential for improving assumptions about variation in species size for other applications of biomass estimates.

## 5 CONCLUSIONS

Our drone method performed well. It estimated plant dry weight biomass more effectively than existing methods used in ecological surveys. This technique appears to be applicable to similar vegetation species in ecosystems with similar canopy structures worldwide. We found the drone method to be most reliable for plants with dense, compact growth forms and least reliable for plants with diffuse growth forms and fine branches. It is important to test method effectiveness against traditional high precision methods as we have done to ensure that the technique delivers useful data. We expect the accuracy, popularity, and applicability of the drone method to improve with technology. We have calculated that limitations of time inefficiencies (relative to simple dimension measurement) should diminish. This new method will improve existing estimates of plant biomass and could address the shortfall in monitoring biomass change across large areas over long time frames by increasing data collection efficiency.

## CONFLICT OF INTEREST

The authors declare that the research was conducted in the absence of any relationships that could be construed as a potential conflict of interest. The funding body had no influence in the survey design, data analysis, interpretation, or decision to publish the results.

## AUTHOR CONTRIBUTIONS

**Justin A. McCann:** Conceptualization (equal); Data curation (lead); Formal analysis (lead); Investigation (lead); Methodology (lead); Project administration (lead); Visualization (lead); Writing – original draft (lead); Writing – review & editing (equal). **David A. Keith:** Conceptualization (equal); Writing – review & editing (equal). **Richard T. Kingsford:** Conceptualization (equal); Formal analysis (supporting); Methodology (supporting); Project administration (supporting); Supervision (lead); Writing – review & editing (equal).

## Supporting information

Appendix

## Data Availability

Data are available on the Dryad Digital Repository at: https://doi.org/10.5061/dryad.xwdbrv1g1.

## References

[ece38891-bib-0001] Anderson, K. , & Gaston, K. J. (2013). Lightweight unmanned aerial vehicles will revolutionize spatial ecology. Frontiers in Ecology and the Environment, 11, 138–146. 10.1890/120150

[ece38891-bib-0002] Belovsky, G. E. , Botkin, D. B. , Crowl, T. A. , Cummins, K. W. , Franklin, J. F. , Hunter, M. L. , Joern, A. , Lindenmayer, D. B. , MacMAHON, J. A. , Margules, C. R. , & Scott, J. M. (2004). Ten suggestions to strengthen the science of ecology. BioScience, 54(4), 345–351.

[ece38891-bib-0003] Berner, L. T. , Jantz, P. , Tape, K. D. , & Goetz, S. J. (2018). Tundra plant aboveground biomass and shrub dominance mapped across the North Slope of Alaska. Environmental Research Letters, 13(3), 35002. 10.1088/1748-9326/aaaa9a

[ece38891-bib-0004] Bonham, C. D. (1989). Measurements of terrestrial vegetation. John Wiley & Sons.

[ece38891-bib-0005] Bradstock, R. A. , & Gill, A. M. (1993). Fire in semiarid, mallee shrublands‐size of flames from discrete fuel arrays and their role in the spread of fire. International Journal of Wildland Fire, 3(1), 3–12. 10.1071/WF9930003

[ece38891-bib-0006] Brandis, K. J. , Kingsford, R. T. , Ren, S. , & Ramp, D. (2011). Crisis water management and ibis breeding at Narran Lakes in arid Australia. Environmental Management, 48(3), 489–498. 10.1007/s00267-011-9705-5 21667315

[ece38891-bib-0007] Brown, S. , Clarke, M. , & Clarke, R. (2009). Fire is a key element in the landscape‐scale habitat requirements and global population status of a threatened bird: the Mallee Emu‐wren (*Stipiturus mallee*). Biological Conservation, 142(2), 432–445. 10.1016/j.biocon.2008.11.005

[ece38891-bib-0008] Capon, S. J. (2003). Plant community responses to wetting and drying in a large arid floodplain. River Research and Applications, 19(5–6), 509–520. 10.1002/rra.730

[ece38891-bib-0009] Capon, S. J. , James, C. S. , Williams, L. , & Quinn, G. P. (2009). Responses to flooding and drying in seedlings of a common Australian desert floodplain shrub: Muehlenbeckia florulenta Meisn. (tangled lignum). Environmental and Experimental Botany, 66(2), 178–185. 10.1016/j.envexpbot.2009.02.012

[ece38891-bib-0010] Catchpole, W. R. , & Wheeler, C. J. (1992). Estimating plant biomass: a review of techniques. Australian Journal of Ecology, 17(2), 121–131. 10.1111/j.1442-9993.1992.tb00790.x

[ece38891-bib-0011] Catford, J. A. , Roberts, J. , Capon, S. J. , Froend, R. H. , Windecker, S. M. , & Douglas, M. M. (2017). Wetland vegetation of inland Australia. In D. A. Keith (Ed.), Australian vegetation (3rd ed., pp. 490–515). Cambridge University Press.

[ece38891-bib-0012] CloudCompare (2018). Version 2.8.1. Available from: http://www.cloudcompare.org/

[ece38891-bib-0013] Cruzan, M. B. , Weinstein, B. G. , Grasty, M. R. , Kohrn, B. F. , Hendrickson, E. C. , Arredondo, T. M. , & Thompson, P. G. (2016). Small unmanned aerial vehicles (micro‐UAVs, drones) in plant ecology. Applications in Plant Sciences, 4(9), 1600041. 10.3732/apps.1600041

[ece38891-bib-0014] Cunliffe, A. M. , Anderson, K. , Boschetti, F. , Brazier, R. E. , Graham, H. A. , Myers‐Smith, I. H. , Astor, T. , Boer, M. M. , Calvo, L. G. , Clark, P. E. , Cramer, M. D. , Encinas‐Lara, M. S. , Escarzaga, S. M. , Fernández‐Guisuraga, J. M. , Fisher, A. G. , Gdulová, K. , Gillespie, B. M. , Griebel, A. , Hanan, N. P. , … Wojcikiewicz, R. (2021). Global application of an unoccupied aerial vehicle photogrammetry protocol for predicting aboveground biomass in non‐forest ecosystems. Remote Sensing in Ecology and Conservation, 8(1), 57–71. 10.1002/rse2.228

[ece38891-bib-0015] Cunliffe, A. M. , Brazier, R. E. , & Anderson, K. (2016). Ultra‐fine grain landscape‐scale quantification of dryland vegetation structure with drone‐acquired structure‐from‐motion photogrammetry. Remote Sensing of Environment, 183, 129–143. 10.1016/j.rse.2016.05.019

[ece38891-bib-0016] Cunliffe, A. , Cunliffe, A. , & Anderson, K. (2019). Measuring above‐ground biomass with drone photogrammetry: Data collection protocol. Protocol Exchange, Version 1 (pp. 1– 16). 10.1038/protex.2018.134

[ece38891-bib-0017] Dandois, J. P. , & Ellis, E. C. (2010). Remote sensing of vegetation structure using computer vision. Remote Sensing, 2(4), 1157–1176. 10.3390/rs2041157

[ece38891-bib-0018] Eldridge, D. J. , & Delgado‐Baquerizo, M. (2017). Continental‐scale impacts of livestock grazing on ecosystem supporting and regulating services. Land Degradation & Development, 28(4), 1473–1481. 10.1002/ldr.2668

[ece38891-bib-0019] Ferrier, S. (2012). Big‐picture assessment of biodiversity change: scaling up monitoring without selling out on scientific rigour. In D. Lindenmayer & P. Gibbons (Eds.), Biodiversity monitoring in Australia (pp. 63–70). CSIRO Publishing.

[ece38891-bib-0020] Freestone, F. L. , Brown, P. , Campbell, C. J. , Wood, D. B. , Nielsen, D. L. , & Henderson, M. W. (2017). Return of the lignum dead: resilience of an arid floodplain shrub to drought. Journal of Arid Environments, 138, 9–17. 10.1016/j.jaridenv.2016.11.011

[ece38891-bib-0021] Friedel, M. H. , Sparrow, A. D. , Kinloch, J. E. , & Tongway, D. J. (2003). Degradation and recovery processes in arid grazing lands of central Australia. Part 2: Vegetation. Journal of Arid Environments, 55(2), 327–348. 10.1016/S0140-1963(03)00026-0

[ece38891-bib-0022] Gross, J. W. , & Heumann, B. W. (2016). A statistical examination of image stitching software packages for use with unmanned aerial systems. Photogrammetric Engineering & Remote Sensing, 82(6), 419–425. 10.14358/PERS.82.6.419

[ece38891-bib-0023] Hunter, J. T. , & Hunter, V. H. (2016). Vegetation of naree and yantabulla stations on the cuttaburra creek, Far North Western Plains, New South Wales. Cunninghamia, 16, 65–100. 10.7751/cunninghamia.2016.16.008

[ece38891-bib-0024] Kankare, V. , Holopainen, M. , Vastaranta, M. , Puttonen, E. , Yu, X. , Hyyppä, J. , Vaaja, M. , Hyyppä, H. , & Alho, P. (2013). Individual tree biomass estimation using terrestrial laser scanning. ISPRS Journal of Photogrammetry and Remote Sensing, 75, 64–75. 10.1016/j.isprsjprs.2012.10.003

[ece38891-bib-0025] Karpina, M. , Jarząbek‐Rychard, M. , Tymków, P. , Borkowski, A. , Tymków, P. , & Borkowski, A. (2016). UAV‐based automatic tree growth measurement for biomass estimation. International Archives of the Photogrammetry, Remote Sensing and Spatial Information Sciences, 41, 685–688. 10.5194/isprsarchives-XLI-B8-685-2016

[ece38891-bib-0026] Keith, D. A. (2004). Ocean shores to desert dunes: the native vegetation of New South Wales and the ACT. Department of Environment and Conservation.

[ece38891-bib-0027] Kumar, L. , & Mutanga, O. (2017). Remote sensing of above‐ground biomass. Remote Sensing, 9, 1–8. 10.3390/rs9090935

[ece38891-bib-0028] Lu, D. (2006). The potential and challenge of remote sensing‐based biomass estimation. International Journal of Remote Sensing, 27(7), 1297–1328. 10.1080/01431160500486732

[ece38891-bib-0029] Lu, D. , Chen, Q. , Wang, G. , Liu, L. , Li, G. , & Moran, E. (2016). A survey of remote sensing‐based aboveground biomass estimation methods in forest ecosystems. International Journal of Digital Earth, 9(1), 63–105. 10.1080/17538947.2014.990526

[ece38891-bib-0030] McIntyre, S. , Cunningham, R. B. , Donnelly, C. F. , & Manning, A. D. (2015). Restoration of eucalypt grassy woodland: effects of experimental interventions on ground‐layer vegetation. Australian Journal of Botany, 62(7), 570–579. 10.1071/BT14246

[ece38891-bib-0031] Menkhorst, P. W. , & Bennett, A. F. (1990). Vertebrate fauna of mallee vegetation in southern Australia. In J. C. Noble , P. J. Joss , & G. K. Jones (Eds.), The mallee lands: A conservation perspective (pp. 39–53). CSIRO Australia.

[ece38891-bib-0032] Moukomla, S. , Srestasathiern, P. , Siripon, S. , Wasuhiranyrith, R. , & Kooha, P. (2018). Estimating above ground biomass for eucalyptus plantation using data from unmanned aerial vehicle imagery. Remote Sensing for Agriculture, Ecosystems, and Hydrology, 1078308, 6. 10.1117/12.2323963

[ece38891-bib-0033] Nichols, J. D. , & Williams, B. K. (2006). Monitoring for conservation. Trends in Ecology and Evolution, 21(12), 668–673. 10.1016/j.tree.2006.08.007 16919361

[ece38891-bib-0034] Nie, X. , Yang, Y. , Yang, L. , & Zhou, G. (2016). Above‐and belowground biomass allocation in shrub biomes across the northeast Tibetan Plateau. PLoS One, 11(4), e0154251. 10.1371/journal.pone.0154251 27119379 PMC4847786

[ece38891-bib-0035] Peng, D. , Zhang, H. , Liu, L. , Huang, W. , Huete, A. R. , Zhang, X. , Wang, F. , Yu, L. E. , Xie, Q. , Wang, C. , Luo, S. , Li, C. , & Zhang, B. (2019). Estimating the aboveground biomass for planted forests based on stand age and environmental variables. Remote Sensing, 11(19), 2270. 10.3390/rs11192270

[ece38891-bib-0036] Pérez‐Harguindeguy, N. , Díaz, S. , Garnier, E. , Lavorel, S. , Poorter, H. , Jaureguiberry, P. , & Cornelissen, J. H. C. (2013). New handbook for standardized measurement of plant functional traits worldwide. Australian Journal of Botany, 61(34), 167–234. 10.1071/BT12225

[ece38891-bib-0037] Pix4D SA . (2018). Pix4Dmapper Pro. Available from: https://www.pix4d.com/

[ece38891-bib-0038] Proulx, R. , Rheault, G. , Bonin, L. , Roca, I. T. , Martin, C. A. , Desrochers, L. , & Seiferling, I. (2015). How much biomass do plant communities pack per unit volume? PeerJ, 3, e849. 10.7717/peerj.849 25802814 PMC4369330

[ece38891-bib-0039] R Core Team . (2018). R: A language and environment for statistical computing. R Foundation for Statistical Computing.

[ece38891-bib-0040] Reiter, N. , Weste, G. , & Guest, D. (2004). The risk of extinction resulting from disease caused by *Phytophthora cinnamomi* to endangered, vulnerable or rare plant species endemic to the Grampians, western Victoria. Australian Journal of Botany, 52(3), 425–433. 10.1071/BT03130

[ece38891-bib-0041] Shendryk, I. , Broich, M. , Tulbure, M. G. , & Alexandrov, S. V. (2016). Bottom‐up delineation of individual trees from full‐waveform airborne laser scans in a structurally complex eucalypt forest. Remote Sensing of Environment, 173, 69–83. 10.1016/j.rse.2015.11.008

[ece38891-bib-0042] Silva, C. A. , Crookston, N. L. , Hudak, A. T. , Vierling, L. A. , Klauberg, C. , & Cardil, A. (2017). rLiDAR: LiDAR data processing and visualization. Available from: https://cran.r‐project.org/package=rLiDAR

[ece38891-bib-0043] Stone, C. , & Coops, N. C. (2004). Assessment and monitoring of damage from insects in Australian eucalypt forests and commercial plantations. Australian Journal of Entomology, 43(3), 283–292. 10.1111/j.1326-6756.2004.00432.x

[ece38891-bib-0044] Suganuma, H. , Abe, Y. , Taniguchi, M. , Tanouchi, H. , Utsugi, H. , Kojima, T. , & Yamada, K. (2006). Stand biomass estimation method by canopy coverage for application to remote sensing in an arid area of Western Australia. Forest Ecology and Management, 222(1–3), 75–87. 10.1016/j.foreco.2005.10.014

[ece38891-bib-0045] Torres‐Sánchez, J. , López‐Granados, F. , Serrano, N. , Arquero, O. , & Peña, J. M. (2015). High‐throughput 3‐D monitoring of agricultural‐tree plantations with unmanned aerial vehicle (UAV) technology. PLoS One, 10(6), e0130479. 10.1371/journal.pone.0130479 26107174 PMC4479442

[ece38891-bib-0046] Verschuyl, J. , Clark, L. , & Loehle, C. (2018). Predicting shrub biomass and current annual growth from field measurements in the Oregon Coast Range. Northwest Science, 92(1), 9–17. 10.3955/046.092.0103

[ece38891-bib-0047] Wallace, L. , Hillman, S. , Reinke, K. , & Hally, B. (2017). Non‐destructive estimation of above‐ground surface and near‐surface biomass using 3D terrestrial remote sensing techniques. Methods in Ecology and Evolution, 8(11), 1607–1616. 10.1111/2041-210X.12759

[ece38891-bib-0048] Westcott, V. C. , Enright, N. J. , Miller, B. P. , Fontaine, J. B. , Lade, J. C. , & Lamont, B. B. (2014). Biomass and litter accumulation patterns in species‐rich shrublands for fire hazard assessment. International Journal of Wildland Fire, 23(6), 860–871. 10.1071/WF13006

[ece38891-bib-0049] Wright, B. R. , & Clarke, P. J. (2007). Fire regime (recency, interval and season) changes the composition of spinifex (Triodia spp.) dominated desert dunes. Australian Journal of Botany, 55(7), 709–724. 10.1071/BT06240

[ece38891-bib-0050] Yates, C. J. , Gosper, C. R. , Hopper, S. D. , Keith, D. A. , Prober, S. M. , & Tozer, M. G. (2017). Mallee woodlands and shrublands: the mallee, muruk/muert and maalok vegetation of southern Australia. In D. A. Keith (Ed.), Australian vegetation (3rd ed., pp. 570–598). Cambridge University Press.

[ece38891-bib-0051] Yelenik, S. G. , & D’Antonio, C. M. (2013). Self‐reinforcing impacts of plant invasions change over time. Nature, 503(7477), 517–520. 10.1038/nature12798 24256723

[ece38891-bib-0052] Zolkos, S. G. , Goetz, S. J. , & Dubayah, R. (2013). A meta‐analysis of terrestrial aboveground biomass estimation using lidar remote sensing. Remote Sensing of Environment, 128, 289–298. 10.1016/j.rse.2012.10.017

