# Supplementary material for: Measuring plant biomass remotely using drones in arid landscapes
Source: Ecol Evol. 2022 May 13;12(5):e8891. doi: 10.1002/ece3.8891 (PMC9106562; doi:10.1002/ece3.8891)
Supplement: Supplementary file 1 — Appendix [file ECE3-12-e8891-s001.docx]

# Appendices
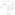


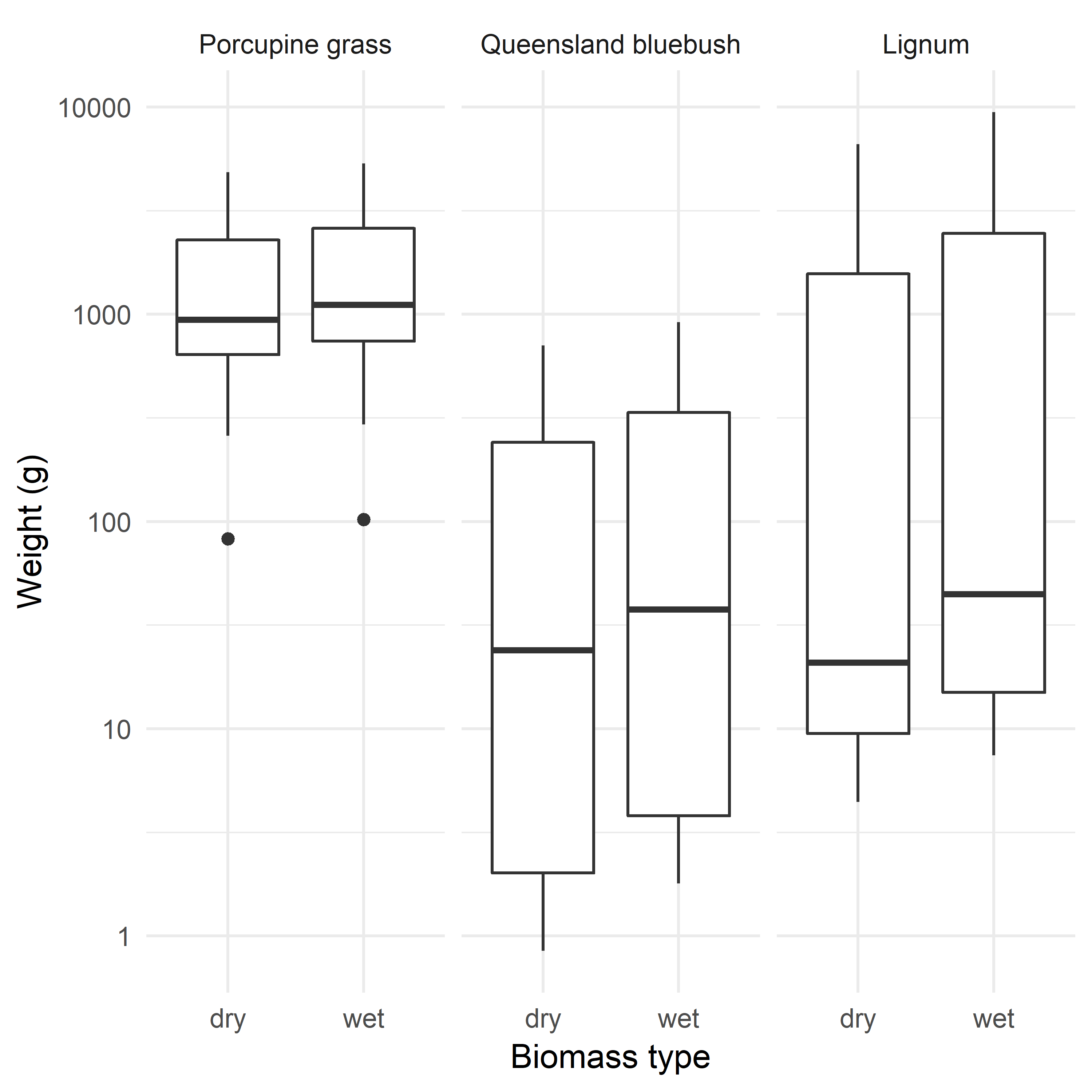


Figure S1. Comparison of dry and wet weight biomass for all plants of the three species of interest (n = 27), measured before and after oven drying in the laboratory.


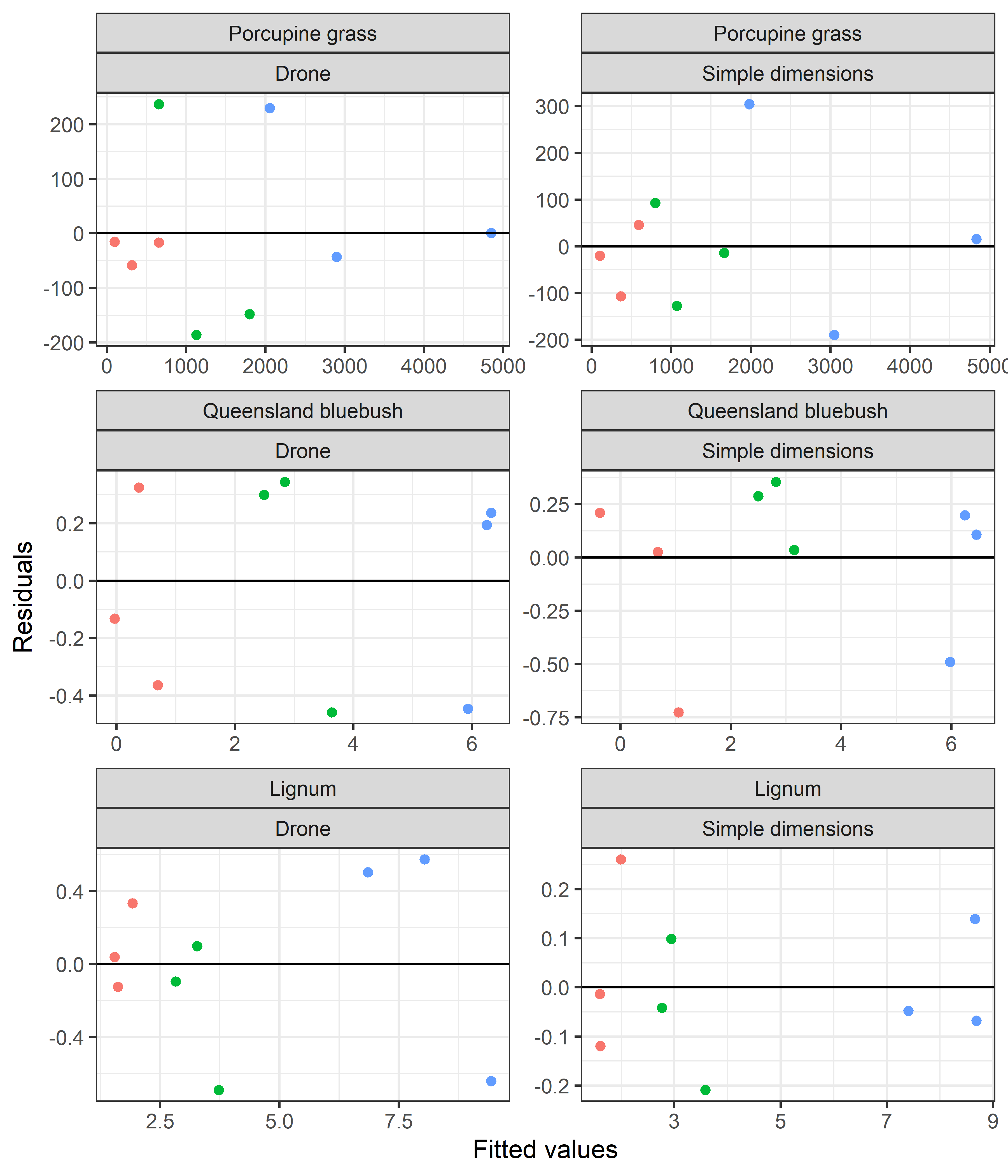


Figure S2. Comparison of residuals vs fitted values (logged) from linear models between dry weight biomass and simple dimension and drone measures for three semi-arid plant species, porcupine grass, lignum and Queensland bluebush and three size classes: small (red), medium (green) and large (blue).
